# Multiomics Analysis Identifies Prognostic Signatures for Sepsis-Associated Hepatocellular Carcinoma in Emergency Medicine

**DOI:** 10.1155/2024/1999820

**Published:** 2024-10-10

**Authors:** Xin Chu, Qi Wu, Linglin Kong, Qiang Peng, Junhua Shen

**Affiliations:** ^1^Department of Emergency, The Second Affiliated Hospital of Nantong University, Nantong, Jiangsu 226001, China; ^2^Department of Infectious Disease, The Second Affiliated Hospital of Nantong University, Nantong, Jiangsu 226001, China

## Abstract

**Objectives:**

Sepsis, caused by the body's response to infection, poses a life-threatening condition and represents a significant global health challenge. Characterized by dysregulated immune response to infection, sepsis may lead to organ dysfunction and failure, ultimately resulting in high mortality rates. The liver plays a crucial role in sepsis, yet the role of differentially expressed genes in septic patients remains unclear in hepatocellular carcinoma (HCC). In this study, we aim to investigate the significance of differentially expressed genes related to sepsis in the occurrence and prognosis of tumors in HCC.

**Methods:**

We conducted analyses by obtaining gene transcriptome data and clinical data of HCC cases from The Cancer Genome Atlas (TCGA). Furthermore, we obtained transcriptomic sequencing results of septic patients from the Gene Expression Omnibus (GEO) database, identified intersecting differentially expressed genes between the two, and performed survival analysis on the samples using LASSO and Cox regression analysis. Combining analyses of tumor mutation burden (TMB) and immune function, we further elucidated the mechanisms of sepsis-related genes in the prognosis and treatment of HCC.

**Results:**

We established a prognostic model consisting of four sepsis-related genes: KRT20, PAEP, CCR3, and ANLN. Both the training and validation sets showed excellent outcomes in the prognosis of tumor patients, with significantly longer survival times observed in the low-risk group based on this model compared to the high-risk group. Furthermore, analyses, such as differential analysis of tumor mutation burden, immune function analysis, GO/KEGG pathway enrichment analysis, and drug sensitivity analysis, also demonstrated the potential mechanisms of action of sepsis-related genes.

**Conclusions:**

Models constructed based on sepsis-related genes have shown excellent predictive ability in prognosis and differential analysis of drug sensitivity among tumor patients. These predictive models can enhance patient prognosis and inform the creation of early treatment protocols for sepsis, consequently aiding in the prevention of sepsis-induced HCC development through the modulation of the overall immune status. This may play a crucial role in patient management and immunotherapy, providing valuable reference for subsequent research.

## 1. Introduction

Hepatocellular carcinoma (HCC) is a common and serious liver cancer condition that typically manifests noticeable symptoms only in the later stages of tumor formation and progresses rapidly [1, 2]. Major risk factors for HCC include chronic viral hepatitis (especially hepatitis B virus infection), alcohol abuse, fatty liver disease, genetic factors, dietary habits, and environmental exposure [3]. Particularly in some developing countries, hepatitis B virus infection is the primary causative factor. With the advancement of scientific research, there is an increasing focus on exploring tumor occurrence and development from a genetic perspective [4, 5]. Differential gene expression plays a significant role in the development of HCC. With the development of transcriptomics and bioinformatics technologies, we have been able to identify differential gene expression patterns between HCC tissues and normal liver tissues [6]. These differential genes not only serve as diagnostic markers for HCC but may also be involved in regulating biological processes such as tumor cell proliferation, metastasis, and apoptosis [7]. However, it remains unclear how these differential genes influence tumor immune responses and immune evasion in HCC.

The liver, as one of the largest visceral organs in the human body, plays a crucial role in physiological and immune functions [8]. However, due to the liver's unique position in metabolism, detoxification, and immune regulation, it is susceptible to various external and internal factors, thereby increasing the risk of developing HCC [9]. Moreover, sepsis, a severe disease caused by systemic infection, is closely associated with liver function. Therefore, the liver, acting as a bridge, further strengthens the relationship between HCC and sepsis [10, 11]. In the fields of oncology and immunology, the interactions among HCC, sepsis, tumor immunity, and differential gene expression are increasingly being scrutinized [12]. The occurrence of sepsis may also accelerate the progression of HCC by inducing inflammatory responses, immune suppression, and metabolic abnormalities, thereby promoting tumor growth and metastasis, ultimately increasing the mortality rate of patients [13, 14].

Acute sepsis is a systemic inflammatory response syndrome, usually caused by bacterial infection. Severe sepsis can lead to multiorgan dysfunction and even endanger life. As the largest immune organ of the human body, the liver plays an important role in the occurrence and development of sepsis [15, 16]. HCC is a malignant tumor. The incidence rate is closely related to cirrhosis, which brings long-term health challenges to patients. The long-term prognosis of patients with acute sepsis and HCC is often influenced by multiple factors. The basic health status and immune system function of patients play a crucial role in prognosis [17, 18]. Patients with underlying diseases or immune suppression are more prone to infections, multiple organ dysfunction, and recurrence. Secondly, the timeliness and effectiveness of treatment are also crucial for prognosis, and postoperative rehabilitation and lifestyle management of patients also have a significant impact on long-term prognosis. The genetic factors and lifestyle habits of patients can also have an impact on the long-term prognosis of acute sepsis and HCC, and some genetic variations may make patients more susceptible to acute sepsis or HCC [19, 20]. In patients with a history of acute sepsis, the likelihood of developing HCC in the later stages may increase, which may be the result of the combined effects of factors such as the immune status and functional recovery of the liver during the infection process [21].

**Figure 1:**
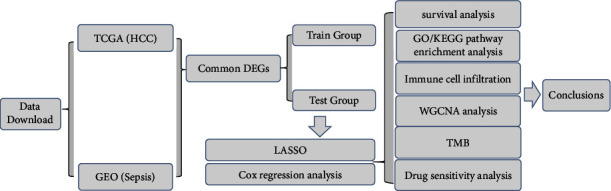
Flowchart of the entire study. This figure shows the sources of our data as well as the main analytical processes.

This study selected differential genes related to sepsis from TCGA and GEO databases, combined with clinical information of patients in these databases, to construct a prognostic model based on these common differential genes. Furthermore, the study analyzed the association between the results of the model and mutation status, immune function, and drug sensitivity response in HCC patients. Pathway enrichment analysis was also conducted to explore potential mechanisms. This study aims to systematically investigate the molecular mechanisms related to differential gene expression, immunity, and sepsis in HCC patients by comprehensively utilizing transcriptomics, bioinformatics, and clinical data, with the goal of providing new scientific evidence for individualized treatment and prognosis assessment of HCC. The whole study is shown in Figure 1.

## 2. Methods

### 2.1. Data Download and Organization

RNA sequencing data of HCC samples and clinical characteristics of patients were obtained from The Cancer Genome Atlas (TCGA) database (374 tumor samples of HCC, 50 adjacent normal samples), while RNA sequencing data of sepsis samples were obtained from the GEO database (GSE6535) (control patients: 17, sepsis patients: 55). The study included seventy-two critically ill patients admitted to the intensive care unit (ICU) of Nepean Hospital, Sydney, Australia. Of these, fifty-five patients were diagnosed to have sepsis, as confirmed by microbiological culture. The remaining seventeen patients did not have sepsis and were therefore used as controls. Differential genes were extracted from the transcriptomes of tumors and adjacent normal tissues in the TCGA database. Subsequently, differential genes were identified between sepsis samples and control group samples in the GEO database. Next, the common differential genes between these two datasets were obtained as further research targets. The process of data processing included the following: raw data download, probe annotation, missing value complementation, and removal of inter-P differences. This data processing procedure was jointly completed by two professional bioinformatics analysts. Data analysis was performed using R software version 4.2.2, making extensive use of the “limma” package.

### 2.2. Prognostic Modeling

We divided the HCC tumor samples into training and validation sets. LASSO regression analysis was conducted in the training set to obtain representative genes utilizing the “glmnet” package, followed by univariate Cox regression to screen for potential prognostic genes, facilitated by the “survival” package. Genes with significant results (*p* value <0.05) in the Cox analysis were considered potential prognostic genes. In the training cohort, patients were divided into low-risk and high-risk groups, using the median risk score as the cutoff point. Subsequently, the model formula from the training set was applied to validate the samples in the validation set, obtaining the risk value for each sample in the validation set to assess the accuracy of the model.

### 2.3. Survival Analysis

Survival differences between high- and low-risk groups were analyzed, and Kaplan–Meier curves were used to demonstrate and compare the differences in survival rates between the high- and low-risk groups, using the “survival” package. Furthermore, ROC analysis was performed to further evaluate the prognostic ability of gene features with the “timeROC” package. To further validate the accuracy of the model predictions, we conducted survival analysis by grouping samples based on different genders and stages separately to assess the accuracy of our model among different groups. Next, by combining the clinical data of pan-cancer from the TCGA database with the risk values obtained from the samples in our constructed model, we divided the samples into high- and low-risk groups based on the median risk value. Further comparison of the survival differences between the high- and low-risk groups in the progression-free survival state was conducted.

### 2.4. Independent Prognostic Analysis

We conducted univariate and multivariate survival analyses based on the age, gender, stage, and grade of the samples, as well as the risk values obtained from our constructed model, to further examine whether our model can independently predict the prognosis of the samples from other factors and validate the reliability of the model predictions, utilizing the “survival” package.

### 2.5. Analysis of Immune Cell Infiltration and Immune-Related Functions

We employed the CIBERSORT algorithm to conduct immune cell infiltration analysis for each sample in the training set, involving 22 types of immune cells [22]. Through immune-related functional analysis, we further identified immune-related functions that differed between high- and low-risk groups, as well as the correlations between risk values and various immune cells, providing reference for subsequent research.

### 2.6. WGCNA Functional Module Analysis

After organizing and classifying the samples in the data, we conducted WGCNA functional module analysis, clustering the genes in our expression matrix into modules, utilizing the “WGCNA” and “limma” packages We identified genes strongly correlated with tumor occurrence and found target genes included in our model across different modules, further validating the accuracy of the model.

### 2.7. Functional Enrichment Analysis

Combining the risk values of each sample with the gene expression matrix of the samples, we conducted functional enrichment analysis on the differentially expressed genes in the high- and low-risk groups. Through GO/KEGG functional enrichment analysis, utilizing the “org.Hs.eg.db” and “enrichplot” packages, we identified pathways showing differential expression between the high- and low-risk groups. After setting filtering criteria based on the results (*p* < 0.05), we have selected the top 10 pathways enriched in pathways as the next research target.

## 3. TMB

Using tumor mutation burden data from samples in the TCGA database, along with risk values calculated for each sample using our constructed model, we analyzed the differences in mutation burden between high- and low-risk groups. Through survival curves, we further demonstrated the survival differences between samples with high and low mutation burden.

### 3.1. Drug Sensitivity Analysis

Combining the drug sensitivity data files in the database (https://osf.io/c6tfx/files/osfstorage), we scored the drug sensitivity for each sample. Then, combining the risk values of each sample, we analyzed the sensitivity of high- and low-risk groups to different drugs, this analysis was performed using the “oncoPredict” package.

### 3.2. Statistical Analysis

Data analysis was conducted using R version 4.2.2, and the main R packages used in the analysis process were displayed in Supplementary file 1, with results presented as mean ± standard deviation (SD). Statistical evaluations were performed with SPSS software version 26.0 (SPSS Inc., USA). GraphPad software (version 8.0.2) was utilized for the creation and statistical analysis of graphs. A *p* value of less than 0.05 was considered statistically significant.

## 4. Results

### 4.1. Acquisition of Public Differential Genes

**Figure 2:**
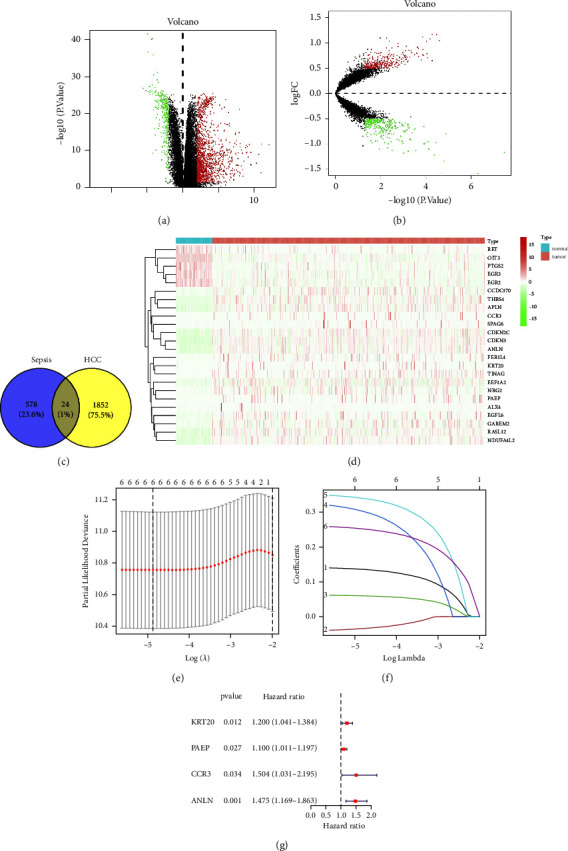
Acquisition of differential genes and prognostic modeling. (a) Volcano plot of differential genes in HCC samples. (b) Volcano plot of differential genes in sepsis samples, with red dots representing upregulated differential genes and green dots representing downregulated differential genes. (c) Intersection of differentially expressed genes, 24 common differentially expressed genes. (d) Heat map of 24 common differentially expressed genes in tumor and normal tissues of HCC samples. (e and f) The result of Lasso regression analysis. It can be seen that modeling 6 genes is more accurate and reliable. (g) The result of Cox regression analysis. The red dot represents high risk, while the green dot represents low risk.

After analyzing the HCC samples in the TCGA database, we obtained differential genes between tumor and adjacent tissues (1876 genes) (Figure 2(a)). After analyzing the sepsis samples in the GEO database, we obtained differential genes between control group samples and sepsis (602 genes) (Figure 2(b)). By intersecting these two sets, we identified 24 common differential genes (Figures 2(c) and 2(d)).

### 4.2. Prognostic Models

After dividing the tumor samples downloaded from the TCGA database into a training set (185 samples) and a validation set (185 samples), we conducted LASSO regression analysis to obtain representative genes, the results of which are shown in Figures 2(e) and 2(f). Univariate Cox regression analysis was performed on the samples in the training set to screen for potential prognostic genes. Finally, a prognostic model was constructed using 4 differential genes related to sepsis (KRT20, PAEP, CCR3, and ANLN), as shown in Figure 2(g). Based on the prognostic model, risk values were obtained for each sample, and patients were divided into low-risk and high-risk groups using the median risk score as the cutoff point.

### 4.3. Survival Analysis

**Figure 3:**
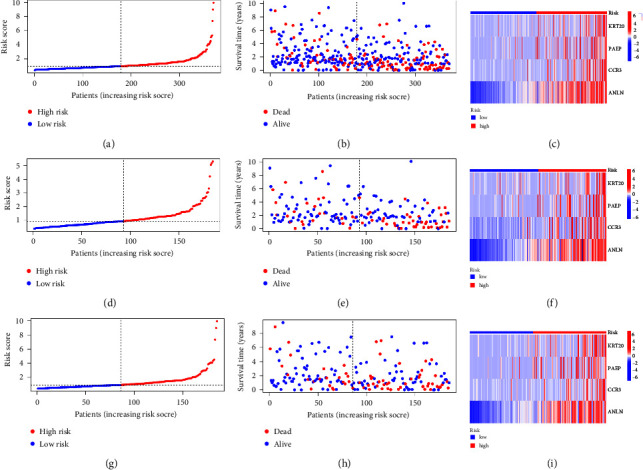
The division of risk for each sample in the prognostic model. (a-c) Risk curve, sample distribution map, and heat map for all sample sets, (d-f) risk curve, sample distribution map, and heat map for training sets, and (g-i) risk curve, sample distribution map, and heat map for validation sets. Red dots indicate low risk or the sample is dead, and blue dots indicate high risk or the sample is alive, *p* < 0.05.

**Figure 4:**
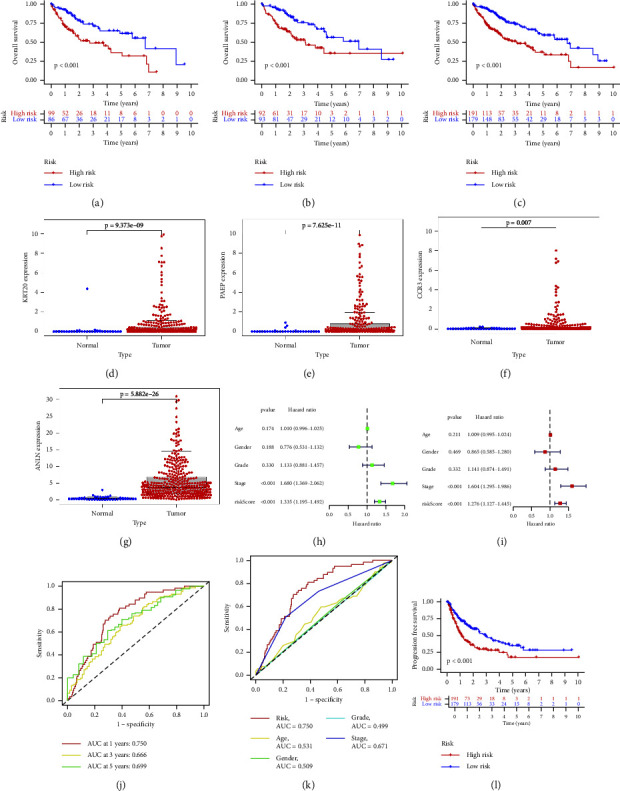
The result of survival analysis and independent prognostic analysis. (a) The survival curves of all samples, (b) the survival curve of the training set, (c) the survival curve of the validation set; horizontal coordinates indicate survival time, vertical coordinates indicate survival rate. (d–g) Differences in expression of 4 modeling genes between tumor and normal tissues. (h) Single-factor prognostic analysis. (i) Multivariate prognostic analysis results. Hazard ratio >1 indicates high factor. (j) ROC curve predicted the survival of the patients at 1, 3, and 5 years. *p* < 0.05. (k) ROC curve of different age groups, genders, stages, and grades, showing that our prognostic model's risk values. Red represents risk score. The larger the area under the curve, the greater the credibility of the results. Risk score is the most accurate prediction result, *p* < 0.05. (l) The result of PFS analysis. Red represents high risk, and blue represents low risk. There is a significant difference in survival time between the high- and low-risk groups in progression-free survival, *p* < 0.05.

Combining the previously obtained risk values for each sample, we employed risk curves for each sample, revealing that patient mortality in the high-risk group increased over time compared to the low-risk group. Moreover, the expression levels of KRT20, PAEP, CCR3, and ANLN were elevated in the high-risk group, as illustrated in Figure 3 (a–c: all samples, d–f: train groups, g–i: test groups). Through survival analysis, it can be observed that, in all samples, training set, and validation set, the survival rate of the low-risk group is higher than that of the high-risk group as time progresses, as shown in Figures 4(a), 4(b), 4(c). Additionally, we further extracted the expression levels of the four genes included in the model, with KRT20, PAEP, CCR3, and ANLN showing higher expression in the high-risk group, as illustrated in Figures 4(d), 4(e), 4(f), 4(g). To further validate the accuracy of the model predictions, we conducted independent prognostic analysis on different age groups, genders, stages, and grades, showing that our prognostic model's risk values have excellent predictive results in both univariate and multivariate prognostic analyses (Figures 4(h) and 4(i)), with *p* values all less than 0.01. Additionally, we further demonstrated the predictive accuracy of the model at 1, 3, and 5 years using ROC curves, The area under the curve (AUC) values were 0.750 at 1 year, 0.666 at 3 years, and 0.669 at 5 years, respectively, underscoring the model's predictive accuracy at these time intervals, as displayed in Figure 4(j). Furthermore, to further validate the accuracy of the model predictions, we used ROC curves to evaluate the accuracy of the model among different age groups, genders, stages, and grades. The results are displayed in Figure 4(k). Combining clinical data from pan-cancer TCGA database and risk values obtained from our model, we divided the samples into high- and low-risk groups based on the median risk value, further comparing the survival differences between the high- and low-risk groups in the progression-free survival status. From the results, we observed significant differences in survival time between the high- and low-risk groups in the progression-free survival period as determined by our model grouping. The results are displayed in Figure 4(l).

### 4.4. Functional Enrichment Analysis

**Figure 5:**
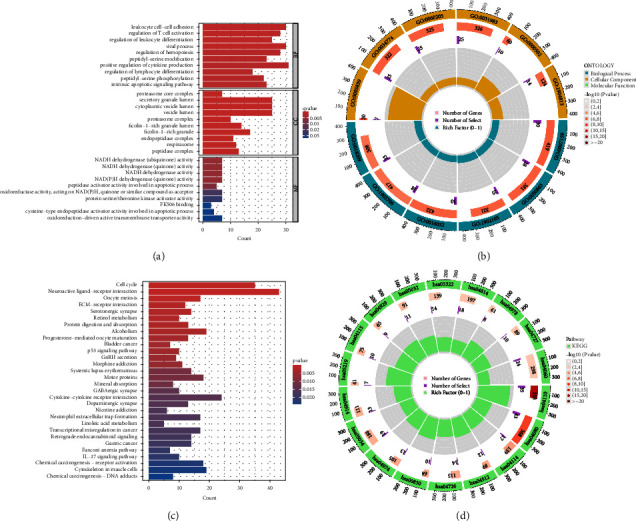
The result of the analysis of functional enrichment analysis. (a) Bar chart of the result of GO enrichment analysis. (b) In this visualization, the outer circle signifies the ID of the GO term, while the subsequent circle conveys the number of genes associated with each GO term. The color intensity of this circle reflects the significance of enrichment, with darker shades indicating higher significance. Additional circles depict the count of coexpressed genes and the heat ratio of genes. (c and d) The outcomes of the KEGG enrichment analysis are displayed through a bar graph where the color gradient represents the *p* values. A shift from lighter to darker hues signifies a gradual increase in *p* values. Moreover, the size of the endpoints on the graph corresponds to the number of genes enriched in the pathway, with larger endpoints indicating a greater abundance of enriched genes.

Through functional enrichment analysis, GO enrichment analysis results indicate that the differential genes in sepsis samples are mainly enriched in the following pathways (BP: leukocyte cell-cell adhesion; regulation of T-cell activation; regulation of leukocyte differentiation; viral process; regulation of hemopoiesis; peptidyl-serine modification; positive regulation of cytokine production; regulation of lymphocyte differentiation; peptidyl-serine phosphorylation; intrinsic apoptotic signaling pathway; CC: proteasome core complex; secretory granule lumen; cytoplasmic vesicle lumen; vesicle lumen; proteasome complex; ficolin-1-rich granule lumen; ficolin-1-rich granule; endopeptidase complex; respirasome; peptidase complex; MF: NADH dehydrogenase (ubiquinone) activity; NADH dehydrogenase (quinone) activity; NADH dehydrogenase activity; NAD(P)H dehydrogenase (quinone) activity; peptidase activator activity involved in apoptotic process; oxidoreductase activity, acting on NAD(P)H, quinone, or similar compound as acceptor; protein serine/threonine kinase activator activity; FK506 binding; cysteine-type endopeptidase activator activity involved in apoptotic process; oxidoreduction-driven active transmembrane transporter activity), as shown in Figures 5(a) and 5(b). To further explore the role of these differential genes in tumorigenesis, we conducted KEGG enrichment analysis on the common differential genes, revealing that these differential genes are mainly enriched in the following pathways (cell cycle; neuroactive ligand-receptor interaction; oocyte meiosis; ECM-receptor interaction; serotonergic synapse; retinol metabolism; protein digestion and absorption; alcoholism; progesterone-mediated oocyte maturation; bladder cancer; p53 signaling pathway; GnRH secretion; morphine addiction; systemic lupus erythematosus; motor proteins; mineral absorption; GABAergic synapse; cytokine-cytokine receptor interaction; dopaminergic synapse; nicotine addiction; neutrophil extracellular trap formation; linoleic acid metabolism; transcriptional misregulation in cancer; retrograde endocannabinoid signaling; gastric cancer; Fanconi anemia pathway; IL-17 signaling pathway; chemical carcinogenesis-receptor activation; cytoskeleton in muscle cells; chemical carcinogenesis-DNA adducts), as shown in Figures 5(c) and 5(d). These enrichment results demonstrate that these differentially expressed genes related to sepsis are indeed significantly enriched in key pathways of tumor development, further emphasizing the significance of studying the intersection between sepsis and HCC.

### 4.5. Immune Cell Infiltration Analysis and Immune-Related Function Analysis

**Figure 6:**
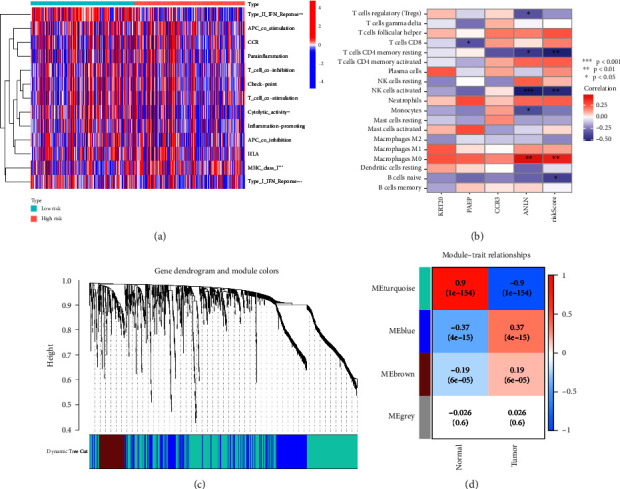
The result of the analysis of immune-related functions and analysis of WGCNA. (a) The result of the analysis of immune-related functions, and the red represents high risk, while the green represents low risk. ^∗^*p*<0.05, ^∗∗^*p*<0.01, ^∗∗∗^*p*<0.001. (b) Correlation analysis between the genes in the model and the immune cells. Red represents a positive correlation, while blue represents a negative correlation. ^∗^*p* < 0.05, ^∗∗^*p* < 0.01, ^∗∗∗^*p* < 0.001. (c) Clustering dendrogram of genes. Various colors represent different modules. (d) Module-trait relationship. Each row represents a module eigengene, and each column represents a trait (normal or tumor). Each cell includes the corresponding correlation and *p* value.

Through immune-related function analysis, it can be seen that the immune-related functions that differ between the high- and low-risk groups are (Type_II_IFN_Reponse, Cytolytic_activity, MHC_class_I, and Type_I_IFN_Reponse), as shown in Figure 6(a). Through immune cell infiltration analysis, it can be observed that the type of immune cells positively correlated with ANLN was macrophage M0, while the type negatively correlated is NK cells activated, T cells regulatory (Tregs), T cells CD4 memory resting, and monocytes, as shown in Figure 6(b). The results of the differences in 22 immune cells in HCC samples are shown in Sfigure 2A and 2B. Combining the risk values of each sample in the prediction model, the differences in immune cell infiltration between high-risk and low-risk groups were compared and analyzed, as shown in Sfigure 2C and 2D.

### 4.6. Analysis of WGCNA Functional Modules

Through WGCNA functional module analysis, our differential genes can be clustered into four modules, among which “MEblue” and “MEbrown” are modules strongly correlated with the tumor group, and KRT20, PAEP, CCR3, and ANLN are located in these two modules, as shown in Figures 6(c) and 6(d). This further confirms the reliability of these common differential genes as predictors in the model.

### 4.7. Differential Analysis of TMB

**Figure 7:**
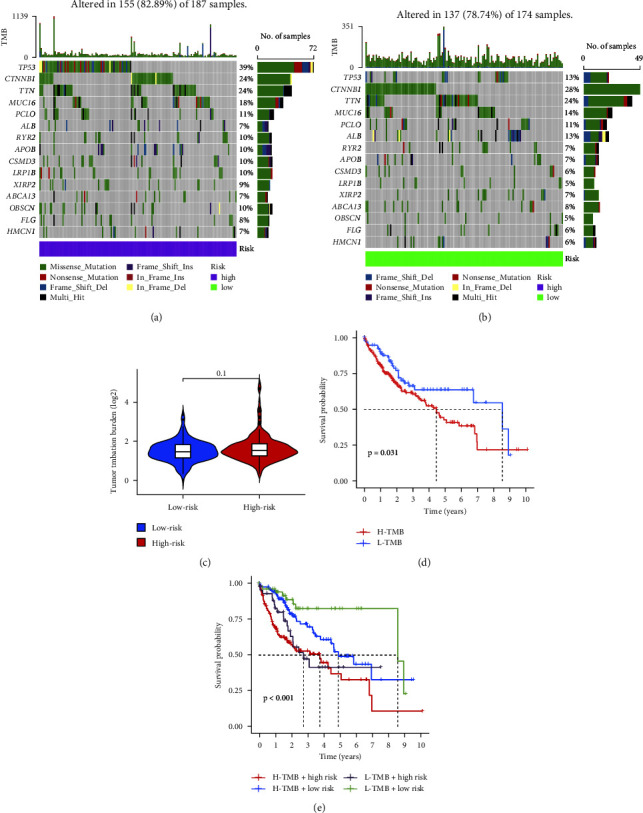
The result of tumor mutation load and survival curve. (a and b) The waterfall plot depicting tumor mutations represents the low-risk group in green and the high-risk group in purple. (c) The differential analysis of tumor mutations assigns blue to the low-risk group and red to the high-risk group. (d) Survival analysis of high and low mutation groups: blue represents the low mutation group, while red represents the high mutation group. (e) Survival analysis of tumor mutation combined with high- and low-risk groups results in four categories: H-TMB + high risk, H-TMB + low risk, L-TMB + high risk, and L-TMB + low risk, with a significance level of *p* < 0.05.

Through the TMB data from samples in the TCGA database, combined with the samples' risk values, we visualized the tumor mutation burden using waterfall plots. The top three mutated genes in the high-risk group were TP53 (39%), CTNNB1 (24%), and TTN (24%), while in the low-risk group, they were CTNNB1 (28%), TTN (24%), and TP53 (13). The results are shown in Figures 7(a) and 7(b). We analyzed the differences in mutation burden between the high- and low-risk groups, and from the results, it can be observed that the mutation frequency in the high-risk group is higher than that in the low-risk group, as depicted in Figure 7(c). Next, we demonstrated the survival differences between high and low mutation groups using survival curves. It can be seen from the results that with increasing time, the survival rate in the high mutation group is lower than that in the low mutation group, as shown in Figure 7(d). To analyze the relationship between mutation burden, risk values, and survival time, we divided the samples into four groups: H-TMB + high risk, H-TMB + low risk, L-TMB + high risk, and L-TMB + low risk. It can be observed from the results that the samples in the L-TMB + low risk group have the highest survival rate as time extends, further indicating the accuracy of our model. The results are shown in Figure 7(e).

### 4.8. Drug Sensitivity Analysis

**Figure 8:**
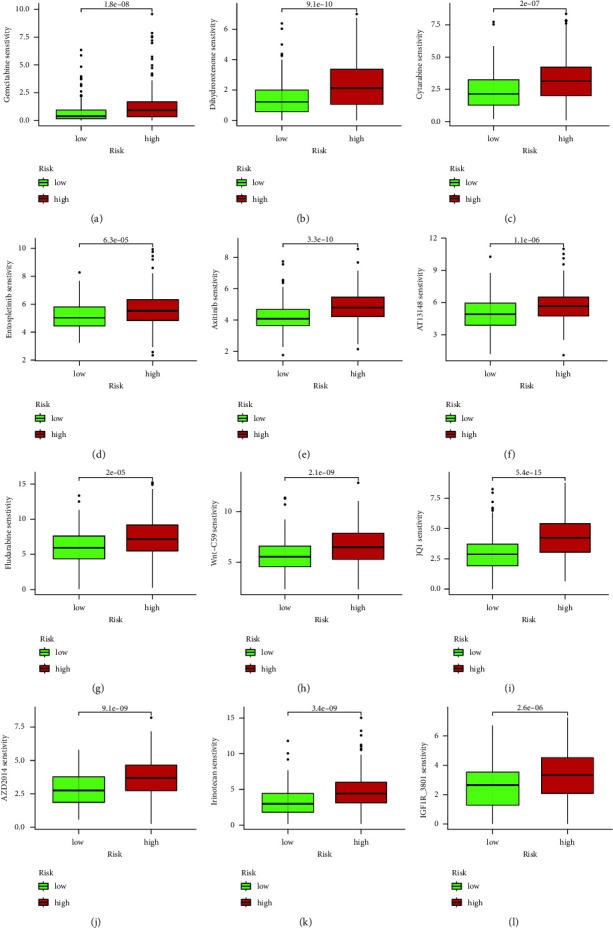
In the graph, the horizontal coordinates represent the risk of the sample, with red indicating high risk and green indicating low risk, while the vertical coordinates represent the sensitivity to the drug. P < 0.05.

Based on the drug sensitivity data file in the database, we scored the drug sensitivity for each sample and analyzed the sensitivity to different drugs in the high-risk and low-risk groups by combining each sample's risk value. Through filtering, we excluded results without differences and obtained 12 drugs with differential drug sensitivity between the high-risk and low-risk groups, as shown in Figures 8(a), 8(b), 8(c), 8(d), 8(e), 8(f), 8(g), 8(h), 8(i), 8(j), 8(k), 8(l). In addition, we obtained 12 drugs with lower sensitivity, as shown in Sfigure 1A–1L.

## 5. Discussion

Hepatocellular carcinoma (HCC) is a highly heterogeneous and immunosuppressive cancer, characterized by complex interactions between tumor cells and the immune microenvironment [23, 24]. Dysregulation of immune responses in HCC leads to tumor progression and limits the efficacy of existing treatment modalities [25, 26]. Understanding the differential gene expression patterns that lead to immune evasion and immune suppression in HCC is crucial for developing targeted immunotherapies and improving patient prognosis [27]. Studies have shown that sepsis can be a potential trigger for hepatocellular carcinoma [28]. The systemic inflammatory response triggered by sepsis may induce abnormal cell proliferation and malignant transformation in the liver, thereby promoting the occurrence and development of hepatocellular carcinoma [20, 29]. Individuals with liver disease are at an increased risk of infection, which is a primary contributor to sepsis. The liver plays a vital role in the immune system, including functions such as pathogen clearance and the regulation of inflammatory responses. However, when hepatic function is compromised, these immune processes may be diminished, rendering patients more susceptible to infections and consequently heightening the risk of sepsis [30]. Previous studies have found that sepsis may directly impair liver function and exacerbate the progression of liver disease.

Sepsis, the systemic inflammatory response caused by sepsis, may lead to liver cell damage and acute deterioration of liver function, thereby accelerating the progression of liver disease, including the occurrence of cirrhosis and hepatocellular carcinoma [31, 32].

The molecular mechanisms of hepatocellular carcinoma (HCC) are complex and multifactorial. They involve gene mutations, abnormalities in cellular signaling pathways, and genetic factors. Typically, these mechanisms include abnormal expression of oncogenes and tumor suppressor genes, abnormal cell proliferation and apoptosis, and changes in the tumor microenvironment. Additionally, chronic viral hepatitis infection and alcohol abuse are significant contributors to HCC. Multiple factors can disrupt the balance between inactivation and activation of tumor suppressor genes and oncogenes, leading to abnormal activation of molecular signaling pathways. Consequently, this is one of the primary reasons why sepsis may lead to the development of HCC [3, 33]. Hepatitis B virus (HBV) and hepatitis C virus (HCV) are the primary causative agents of hepatocellular carcinoma (HCC). Cirrhosis is the consequence of prolonged chronic liver disease, leading to an elevated risk of HCC development. Prolonged alcohol abuse can induce liver damage and elevate the risk of developing HCC. Fatty liver disease, including nonalcoholic fatty liver disease (NAFLD) and nonalcoholic steatohepatitis (NASH), are also recognized as risk factors for HCC [34–36]. Research has demonstrated that aberrant activation of molecules in various signaling pathways regulating cell survival, apoptosis, cell cycle, proliferation, and differentiation contributes to the advancement of HCC. During sepsis, immune cell function, including macrophages and T cells, is inhibited by inflammatory mediators, diminishing the body's capacity to combat infections [37]. Long-term inflammatory response can result in immune system exhaustion and depletion of the body's immune resources, rendering it unable to effectively respond to infections. The inflammatory response triggered by sepsis can cause damage to lymphatic organs and disrupt the normal function of the immune system [38]. Sepsis may disrupt the balance of immune regulation in the body, disrupting the balance between inflammation and anti-inflammatory responses, leading to the emergence of immune suppression [39]. Sepsis can further induce the occurrence of HCC by inducing immune suppression in the body.

As research on the molecular mechanisms of HCC and sepsis progresses, there is increasing attention on exploring the association between them at the genetic level. Exploring their potential biological mechanisms through differential gene analysis and functional analysis methods, analysis of differential genes reveals key genes and signaling pathways driving the onset of HCC and immune dysfunction. These genes play a crucial role in regulating the tumor microenvironment of hepatocellular carcinoma and immune responses [40, 41]. The upregulation of common immune checkpoint molecules (such as PD-L1 and CTLA-4) in HCC leads to T-cell exhaustion and immune evasion [42, 43]. The use of immune checkpoint inhibitors targeting these immune checkpoints has yielded promising results in clinical trials, highlighting the importance of differential gene expression analysis in identifying potential therapeutic targets for HCC.

Prognostic models can guide doctors to promptly initiate treatment in emergency situations by assessing the severity of the patients and predicting the progression of the disease. By analyzing the clinical indicators and biomarkers of patients, prognostic models can provide more accurate assessments of their condition, thereby facilitating doctors to make faster diagnosis and treatment decisions [44]. In cases of acute sepsis, appropriate treatment measures can be taken promptly based on the evaluation results of the prognostic model, thereby reducing the patient's mortality rate and mitigating the impact of acute sepsis on the patient's systemic immune status [45, 46]. The prognostic model also has the potential to facilitate precise medication in acute situations. By integrating patients' physiological indicators, disease characteristics, and genotype information, prognostic models can provide doctors with more accurate medication recommendations, help prevent the overuse and improper use of drugs, and improve treatment effectiveness, thereby further alleviating long-term prognosis and reducing the likelihood of HCC occurrence.

This study applied techniques such as RNA sequencing to tumor tissues and biological specimens from sepsis patients, identifying genes with significantly altered expression levels in HCC and sepsis. Using these differentially expressed genes as a starting point, the study further explored their potential roles in the onset of HCC and patient prognosis. Additionally, through analysis of immune cell infiltration and differences in immune function, the study further confirmed the roles of these common differentially expressed genes, providing a reference for further exploration of the potential association between the two conditions.

However, this study has some limitations. Firstly, due to limitations in sample size and study design, we cannot exclude the influence of other potential factors on differential gene expression, thus requiring larger-scale studies for validation. Secondly, this study is limited to observational research and has not delved into the functions and mechanisms of differential genes and immune factors, necessitating further cellular and animal experiments.

## 6. Conclusions

This study obtained transcriptomic data of HCC tumor and adjacent tissues from TCGA, as well as data from sepsis patients and control groups from the GEO database. Differentially expressed genes were identified in each dataset, and the common differentially expressed genes were further investigated. Using these common differential genes and patient clinical information, a prognostic model was constructed, which showed good predictive performance for HCC. Additionally, significant differences were observed between high- and low-risk groups based on this prognostic model. Furthermore, the analysis of the results from the common differential gene prediction model was related to HCC patient mutation status, immune function, and drug sensitivity reactions, providing a reference for further research on the potential association between sepsis and HCC. These predictive models can enhance patient prognosis and inform the creation of early treatment protocols for sepsis, consequently aiding in the prevention of sepsis-induced HCC development through the modulation of the overall immune status.

## Data Availability

The original data come from TCGA (https://portal.gdc.cancer.gov/) and GEO (https://www.ncbi.nlm.nih.gov/geo/), and the data are accurate.

## Supplementary Materials

Supplementary Materials Sfigure 1: results of drug sensitivity analysis (high risk < low risk). In the graph, the horizontal coordinates represent the risk of the sample, with red indicating high risk and green indicating low risk, while the vertical coordinates represent the sensitivity to the drug. *p* < 0.05. Sfigure 2: the results of scoring the immune microenvironment. A: immune cell infiltration results of 22 immune cells in samples. B: violin plot of 22 immune cell infiltration results, with blue representing normal tissue and red representing tumor tissue. C: immune cell infiltration results of 22 immune cells in high-risk group and low-risk groups. D: boxplot of 22 immune cell infiltration results, with blue representing low-risk groups and red representing high-risk groups, *p* < 0.05. Supplementary file 1: the main R package information. Analysis methods and the main R package information used in the analysis methods.
