# Supplementary material for: Multiomics Analysis Identifies Prognostic Signatures for Sepsis-Associated Hepatocellular Carcinoma in Emergency Medicine
Source: Emerg Med Int. 2024 Oct 10;2024:1999820. doi: 10.1155/2024/1999820 (PMC11486536; doi:10.1155/2024/1999820)
Supplement: Supplementary Materials — Sfigure 1: results of drug sensitivity analysis (high risk < low risk). In the graph, the horizontal coordinates represent the risk of the sample, with red indicating high risk and green indicating low risk, while the vertical coordinates represent the sensitivity to the drug. p < 0.05. Sfigure 2: the results of scoring the immune microenvironment. A: immune cell infiltration results of 22 immune cells in samples. B: violin plot of 22 immune cell infiltration results, with blue representing normal tissue and red representing tumor tissue. C: immune cell infiltration results of 22 immune cells in high-risk group and low-risk groups. D: boxplot of 22 immune cell infiltration results, with blue representing low-risk groups and red representing high-risk groups, p < 0.05. Supplementary file 1: the main R package information. Analysis methods and the main R package information used in the analysis methods. [file 1999820.f1.zip › Supplementary_file_1.pdf]

Supplementary file 1：The main R package information

Analyses	R packages
LASSO regression analysis	"glmnet"
COX regression analysis	'survival"
Survival analysis	"survival";"survminer";"timeROC"
Independent prognostic analysis	survival'
WGCNA Functional Module Analysis	"GO.db"； "preprocessCore"； "impute","limma"； "WGCNA"
Immune-related functional analysis	"BiocManager","limma", "GSVA", "GSEABase", "pheatmap",and "reshape2"； "CIBERSORT"
Functional enrichment analysis	"colorspace";"stringi";"ggplot2";"circlize";"RColorBrewer"; "ggpubr";"org.Hs.eg.db";"DOSE";"clusterProfiler";"enrichplot";"ComplexHeatmap"
Drug sensitivity analysis	"limma", "car", "ridge", "preprocessCore", "genefilter", "sva", "biomaRt","GenomicFeatures", "maftools", " stringr", "org.Hs.eg.db","TxDb.Hsapiens.UCSC.hg19.knownGene"," oncoPredict"
